# Many Local Pattern Texture Features: Which Is Better for Image-Based Multilabel Human Protein Subcellular Localization Classification?

**DOI:** 10.1155/2014/429049

**Published:** 2014-06-24

**Authors:** Fan Yang, Ying-Ying Xu, Hong-Bin Shen

**Affiliations:** ^1^Institute of Image Processing and Pattern Recognition, Shanghai Jiao Tong University, Shanghai 200240, China; ^2^Key Laboratory of Optic-Electronic and Communication, Jiangxi Science & Technology Normal University, Nanchang 330013, China; ^3^Key Laboratory of System Control and Information Processing, Ministry of Education of China, Shanghai 200240, China

## Abstract

Human protein subcellular location prediction can provide critical knowledge for understanding a protein's function. Since significant progress has been made on digital microscopy, automated image-based protein subcellular location classification is urgently needed. In this paper, we aim to investigate more representative image features that can be effectively used for dealing with the multilabel subcellular image samples. We prepared a large multilabel immunohistochemistry (IHC) image benchmark from the Human Protein Atlas database and tested the performance of different local texture features, including completed local binary pattern, local tetra pattern, and the standard local binary pattern feature. According to our experimental results from binary relevance multilabel machine learning models, the completed local binary pattern, and local tetra pattern are more discriminative for describing IHC images when compared to the traditional local binary pattern descriptor. The combination of these two novel local pattern features and the conventional global texture features is also studied. The enhanced performance of final binary relevance classification model trained on the combined feature space demonstrates that different features are complementary to each other and thus capable of improving the accuracy of classification.

## 1. Introduction

During the past two decades, molecular, subcellular, cellular, and supercellular structures are visualized manually by biologists; however, the constantly updated techniques of automated microscopic imaging and biological tissue labeling have created revolutionary development opportunities for those of structure visualization [1]. For instance, each advance in automated microscopic technique can provide biologists with distinct perspectives on functional studies of corresponding living organisms. Take the protein subcellular location prediction (PSLP) as an example, one of the main advantages of automated microscopes is able to collect large amounts of protein subcellular location images with minimal human intervention, which has provided a very nice environment of source data for image-based protein subcellular location prediction (I-PSLP) subsequently. However, there are two inescapable potential problems hiding behind.

The first problem of current situation is big image data. Efficient observation imaging devices, for example, automated brightfield microscopes, confocal microscopy, and so forth, lead to a data explosion accompanied by the arrival of the era of big image data. Tremendous volumes of bioimaging data have been generated in almost every branch of biology and the deluge of high resolution and complicated biological and biomedical images poses significant challenges for the image computing community. Furthermore, the spatial distribution of target protein in a given cell type is critical to understanding protein function and how the cell behaves. But, it is always daunting for even a single cell type to acquire this spatial distribution information, because it is estimated that having a single image for every combination of cell type, protein, and timescale would require the order of 100 billion images [2].

The second is the limited human power. On the existing biological microscope image analysis field, most work of predicting protein subcellular localization has been done through biological experiments and visual inspection to provide crucial information to reveal its corresponding function in the postgenomic era [3]. When concerns the increasing bioimage data, manual and visual notation are slow and expensive, and the datasets of subtle phenotype changes are becoming too large for manual analysis. Even though putting the scales of biomedical image atlas aside, the results from visual analysis are not easily compared between papers or groups because the judgments are often highly variable from one expert to another.

Therefore, it is particularly desired and urgent to construct automated image-based protein subcellular location prediction (AI-PSLP) analysis system with high accuracy and reproducibility better than visual inspection and other manual operations. Some automated data-driven approaches have been constructed during recent years [4, 5]. Interestingly, there is even evidence showing that automated systems can perform better than humans [2, 4]. The reason can be that automated systems are unbiased, while human-based analysis's evaluation may (even unconsciously) be influenced by the desired outcome. High resolution image benchmark, highly discriminative image features, and effective machine learning algorithms can be summarized as the three important components of an AI-PSLP system.

Basically, subcellular location pattern representation in a biological image, for example, immunohistochemistry (IHC) or immunofluorescence (IF) image can be described by a variety of numeric features. Efforts for developing effective image descriptors for AI-PSLP can be generally summarized into the following two categories:

1. studies on global subcellular location features, which can be regarded as global distribution features [6], for example, Haralick features calculated from gray-level cooccurrence matrix of image and DNA features extracted from the distance information between the relative protein and nuclear [7];
2. studies on local texture or structure pattern, which means these kinds of feature descriptors aim to mine and represent local micropatterns of the biological images.

In essence, AI-PSLP is a combination of image processing and pattern recognition. Therefore, many outstanding image processing operators or descriptors can make a contribution to improving the accuracy of AI-PSLP. In recent years, the local texture patterns have attracted much attention in the subcellular location distribution image processing field. This is due to local descriptors offering robustness against rotation and translation in localized regions of images. For example, Nanni and Lumini are two of the pioneer researchers to AI-PSLP by using invariant local binary patterns (LBP) [8], which obtained high classification accuracy. In addition, with the motivation of improving standard LBP, Nanni et al. also considered different shapes for the neighborhood calculation, and the corresponding encoding was employed for subsequent medical image analysis [9]. Furthermore, our group employed local feature, that is, LBP and its two variants, for example, local ternary pattern (LTP) and local quinary pattern (LQP) features [9, 10], to improve the classification accuracy of AI-PSLP [11, 12]. Besides features mentioned above, Coelho et al. has introduced a new feature extraction protocol for fluorescence image classification of subcellular location patterns by using the speeded-up robust features (SURF) [13]. The highlight of this protocol is a generic approach and could be applied to other types of local feature, such as scale-invariant feature transform (SIFT) or any combination of image feature descriptors.

It has not escaped our attention that many other image local texture operators were proposed and applied to the fields of image processing, for example, image retrieval and image classification. Murala et al. proposed a novel image descriptor named local tetra pattern (LTrP) for content-based image retrieval [14]. LTrP can effectively capture consistency of the gradient change in horizontal and vertical direction between the center pixel and its neighbors and then encodes all these tetra patterns as well as its magnitude patterns as the local features of images. Besides, Guo et al. proposed a completed local binary pattern (CLPB) by using local difference sign-magnitude transform (LDSMT) [15], which decomposes the image into two complementary components, namely, the signs and the magnitudes. Moreover, Guo et al. applied CLPB to texture image classification.

In this study, we aim to find out good feature descriptors from multiple local texture patterns on human multilabel subcellular location image classification problem. For this purpose, beyond the global texture image descriptor that already applied in the existing studies, we focus on investigating and comparing the performance of LTrP and CLBP with the standard LBP approach.

In addition to effective image descriptors and high quality image resources, effective algorithms of machine learning are also essential for the comparison purpose. Most classification approaches of AI-PSLP have focused on single-label datasets [7–9, 16, 17]. That is, these kinds of classification systems work under an assumption that each protein belongs to only one subcellular location. However, the real situation is that approximately 20% of human proteins coexist at two or more different subcellular locations [11, 16]. Coincidentally, Stadler et al. showed that even approximately 60% of proteins colocalize to multiple organelles [17].

Therefore, based on the previous multilabel image samples classification work of our group [11], we carried out current work on a more comprehensive large database, which contains 258 single-label proteins and 90 multilabel proteins. The binary relevance (BR) classifier by using a series of support vector machine (SVM) is trained for dealing with the multilabel samples [18].

Our experimental results show the following conclusions. First, the LTrP and CLBP descriptors consistently perform much better than LBP and global features in terms of top features ranked in the feature selection process by stepwise discriminant analysis (SDA). Second, the combination of multiple descriptors will improve multilabel and single-label samples classification accuracies simultaneously.

## 2. Methods

Efforts for developing AI-PSLP can generally be summarized in the following four aspects:

1. benchmark dataset preparation, which means an organized collection of data for subsequent works;
2. image preprocessing level, which includes spatial transformation to bring images to a common reference frame and subsequent image normalization and object separation;
3. image feature level, which includes feature extraction and redundancy removing (feature reduction or selection), and all approaches in this level aim to effectively quantizing the inherent property of original database and then transforming the input data into a reduced representation set of features;
4. classification algorithm level, which focuses on training classifier model and paradigm design.

### 2.1. Dataset

In this study, all images employed to build our benchmark dataset are immunohistochemical (IHC) images from the Human Protein Atlas (HPA, http://www.proteinatlas.org/) [19, 20]. For immunostaining, both off-target binding due to cross-reactivity with other proteins and other artifacts are two important complications; hence, in order to collect high quality dying images, all of the proteins we selected are based on protein evidence level as well as the combination of the validation score and the reliability score, that is, supportive validation and high reliability [21].

Moreover, since human proteins have been demonstrated that can coexist at two or more different subcellular locations in a lot of the literatures [16, 17], we build our dataset consisting of both multilabel and single-label samples. A benchmark of 348 proteins composed of 4,364 IHC images, which involve six major subcellular locations, was chosen from the HPA. The six subcellular locations are cytoplasm, endoplasmic reticulum (ER), golgi apparatus, mitochondria, nucleus, and vesicles. Among the 348 proteins, 7 proteins are with three organelle labels and 83 proteins are with two organelle labels, which are both considered as multilabel samples.

### 2.2. Image Preprocessing Level

Since the IHC images in HPA are stored in RGB mode, to avoid artifacts from poorly stained images, for example, too much cyan dye, we apply spatial transformation to bring images to a common reference frame. Namely, we firstly transform the original IHC images from RGB space to HSV space and then remove images whose hue values exceeded a threshold of 13, the same as [7] and finally transform the images back to RGB space. Besides, the IHC images in HPA are the mixtures of two major components, that is, DNA and protein. These two elements are generally labeled with different colors; that is, the brown regions represent the proteins and the purple regions are the DNA stains in the cell nucleus. Since our concern is the distribution of protein of IHC image, the second problem of preprocessing level we faced is object separation or color separation, that is, to separate the protein channel from that of DNA. In this study, the same approach of color separation used in our previous works, that is, linear separation (LS), was employed to fulfill the separation, and for more details about LS, readers are suggested to read [7, 12].

### 2.3. Image Feature Level

Image features and numerical and fundamental descriptors can be computed from an image to represent its important aspects. More specifically, features can refer to a general neighborhood operation no matter if based on global statistics or local region statistics. Features can be also defined as specific structures in the image itself, for example, simple structures such as points or edges to more complex structures such as textures and objects. The set of features of a given database instance is often grouped into a feature vector. The reason for doing this is that the vector can be treated mathematically. In this study, global and local features are employed and expected to provide complementary information for subsequent classification.

#### 2.3.1. Global Features

The well-established subcellular location features (SLFs) have been extensively demonstrated effective for AI-PSLP [7, 22]. In this study, Haralick feature and DNA feature are employed as the global features. Haralick texture features and DNA distribution features are extracted from the separated protein and DNA channels. Haralick features describe image texture by some intuitive aspects of image, such as inertia and isotropy [22–24]. Here, we used Daubechies wavelet with the vanishing moments from 1~10 (db 1~db 10) to 10 levels decomposition on each db when extracting Haralick features in protein channel. Ten levels decompositions are helpful for deep mining essential characteristics of protein distribution features through successive change of scale parameter of Daubechies wavelet. Hence, a total of 836-dimensional Haralick features of each IHC image are obtained. Moreover, as another global feature, 4-feature components of DNA-protein overlap were also employed because nucleus is fairly consistent among cells as a common point and may provide reference for determining the protein localization pattern [4, 7]. More details of both global features mentioned above can be found in our previous works [11, 12].

#### 2.3.2. Local Pattern Features

Naturally, as a complementary to the global features, local pattern descriptors can well describe local patches or multiple interest regions of images. Many previous studies have shown that the performance of AI-PSLP can be significantly enhanced by incorporating the LBP and its variant local texture patterns. However, it is still not clear whether many other local texture patterns are useful for describing the IHC images and which of them will be more discriminative for describing the multilabel samples. Hence, local binary pattern (LBP), local tetra pattern (LTrP), and completed local binary pattern (CLBP) are employed in order to find out which local texture patterns are better for IHC image description and multilabel human protein subcellular localization classification.

(*1) Local Binary Pattern.* The local binary pattern (LBP) is a type of powerful feature widely used for classification in computer vision, which was proposed by Ojala et al. [25, 26]. As a simple yet efficient operator, LBP applies the combination of local structural model quantization and the concatenate histogram statistics to generate feature vector which can well describe each local interesting region. The standard LBP feature vector is created in the following steps.

**Figure 1:**
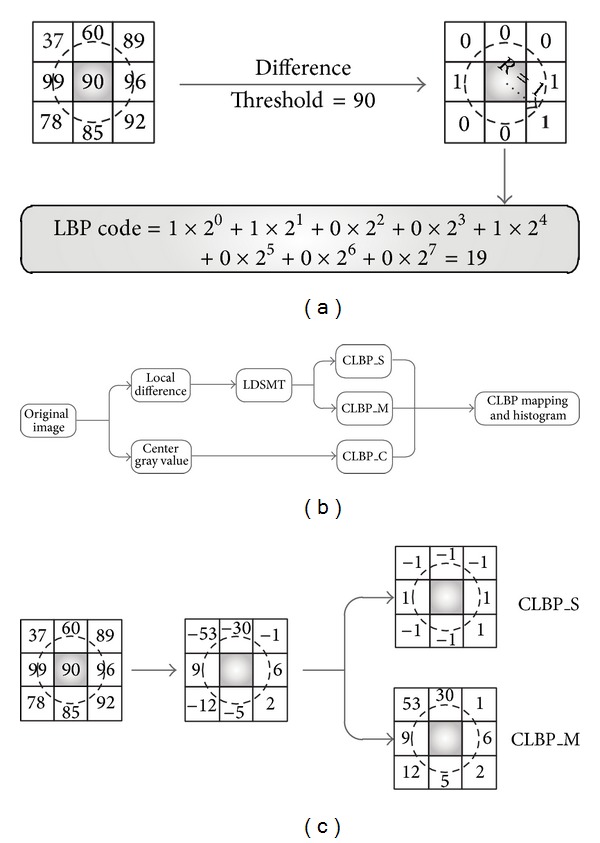
Illustrations of how to calculate LBP and CLBP features for an 8-neighborhood pixels. (a) The standard LBP operator (see (1)). (b) The framework of CLBP operator (see, (3) and (5)). (c) Example to obtain sign and magnitude pattern component from CLPB (see (1) and (4)).

(1) Local region statistics: let each pixel be selected as center pixel and compared to each of its 8-neighbor pixels. Follow the center pixel along a circle, that is, clockwise or counterclockwise.
(2) Binary encoding: the center pixel's value is less than the neighbor's value, output “1”; otherwise, output “0”; see Figure 1(a). This gives an 8-digit binary number. Hence, gray values of all pixels are converted to its corresponding local binary coding. Consider
  (1) $$\begin{array}{c} {\text{LBP}}_{N,R}=\overset{N-1}{\underset{k=0}{\sum }}2^{k}\cdot s\left(g_{k}-g_{c}\right), \end{array}$$

where

(2) $$\begin{array}{c} s\left(x\right)=\{\begin{array}{cc} 1, & x\geq 0\\ 0, & \text{otherwise}. \end{array} \end{array}$$

*N* and *R* represent the number of neighboring pixels and the radius of the neighborhood, respectively, *g*_*c*_ denotes the gray value of the center pixel, and *g*_*k*_ denotes the gray value of neighboring pixel.

(3) Histogram statistics: it converts binary coding to decimal and computes the histogram, traverse all pixels, of the frequency of each “decimal number” occurring. The histogram of the binary patterns obtained over the neighborhood is used for describing the local texture features [27]. We extracted the standard LBP features based on the configurations of *R* = 1 and *N* = 8. LBP can be represented by 256-dimensional features.

The most important properties of the LBP operator are simple computations and powerful illumination invariant while comparing to other descriptors in real-world applications. Furthermore, the LBP operator can be easily adapted to be used together with other image descriptors.

(*2) Completed Local Binary Pattern.* Although LBP feature conveys much discriminant information of local structure, some important information are ignored. Hence, taking effectively representing the missing information into account, Guo et al. proposed a completed local binary pattern named CLPB [15], which decomposes the image into two complementary components, namely, the signs (CLBP_S) and the magnitudes (CLBP_M); see Figures 1(b) and 1(c). Referring to (1), we can simply calculate the difference between *g*_*c*_ and *g*_*k*_; that is, *d*_*k*_ = *g*_*k*_ − *g*_*c*_. The local difference vector [*d*_0_, *d*_1_,…, *d*_*N*−1_] represents the image local structure at center pixel *g*_*c*_. Obviously, *d*_*k*_ can be further decomposed into two components by LDSMT as follows:

(3) $$\begin{array}{c} d_{k}=s_{k}^{\prime }\times m_{k}, \end{array}$$

where *s*_*k*_′ = sign⁡(*g*_*k*_ − *g*_*c*_) is the sign function of *d*_*k*_, only one different from *s*(*x*) defined in (1), *s*_*k*_′ is set to −1 in the case of the value of *d*_*k*_ is less than 0, while *s*(*x*) in (1) is set to 0; otherwise, *s*_*k*_′ is set to −1; *m*_*k*_ = |*g*_*k*_ − *g*_*c*_| is the magnitude function of *d*_*k*_.

Hence, with (3), [*d*_0_, *d*_1_,…, *d*_*N*−1_] is transformed into a sign vector [*s*_0_′, *s*_1_′,…, *s*_*N*−1_′] and a magnitude vector [*m*_0_, *m*_1_,…, *m*_*N*−1_]. It is obvious that CLBP_S operator is the same as the standard LBP operator defined in (1). Since *m*_*k*_ is continuous value instead of binary coding “1” or “−1” like *s*_*k*_′, it is unable to code as that of *s*_*k*_′. Inspired by CLPB_S coding strategy, CLBP_M operator is defined in a consistent form with that of CLPB_S as follows:

(4) $$\begin{array}{c} {\text{CLBP}\_\text{M}}_{N,R}=\overset{N-1}{\underset{k=0}{\sum }}2^{k}\cdot s\left(m_{k}-\tau \right), \end{array}$$

where function *s*(·) is defined in (1) and *τ* is a threshold to be determined adaptively, that is, the mean value of *m*_*k*_ from the original image.

Besides, the center pixel, which expresses the image local gray level, also has discriminant information. To make it consistent with CLBP_S and CLBP_M, the center pixel are defined as CLBP_C by simply encoding using a binary code after global thresholding, which is defined as follows:

(5) $$\begin{array}{c} {\text{CLBP}\_\text{C}}_{N,R}=s\left(g_{c}-c\right), \end{array}$$

where function *s*(·) is defined in (1), and the threshold *c* denotes the average gray value of the whole image.

These three operators, CLBP_S, CLBP_M, and CLBP_C, could be combined in two ways, jointly or hybridly. In this study, we applied 3D joint histogram of them as the local feature to feed into subsequent classifiers since the results of joint approach in [15] are much better than hybridly approach. In order to achieve a fair comparison between CLBP and LBP on effective description, we extracted CLBP features based on the same configurations of LBP; that is, *R* = 1 and *N* = 8.

It is worth pointing out that the CLBP features are extracted based on uniform and rotation invariant pattern mapping, while the LBP features without pattern mapping are used as a baseline to be compared with. Since 256 patterns could be mapped to 10 patterns via uniform and rotation invariant mapping, the CLBP after concatenating 3D joint histogram is 200 (10 ∗ 10 ∗ 2) dimensions in total, which means the dimensions between CLBP and LBP are closest.

(*3) Local Tetra Pattern*. Local tetra pattern (LTrP) descriptor was proposed by Murala et al. [14]. It binary encodes the relationship between the center (or referenced) pixel and its neighbors characterized by transformation consistency statistics of directional derivative in horizontal and vertical direction. Then binary encodes the magnitudes of center pixel. Finally converts both binary codes to decimal and computes the histogram traversing all pixels [14]. Basically, LTrP descriptor is made up of two parts, that is, tetra pattern and magnitude pattern.

**Figure 2:**
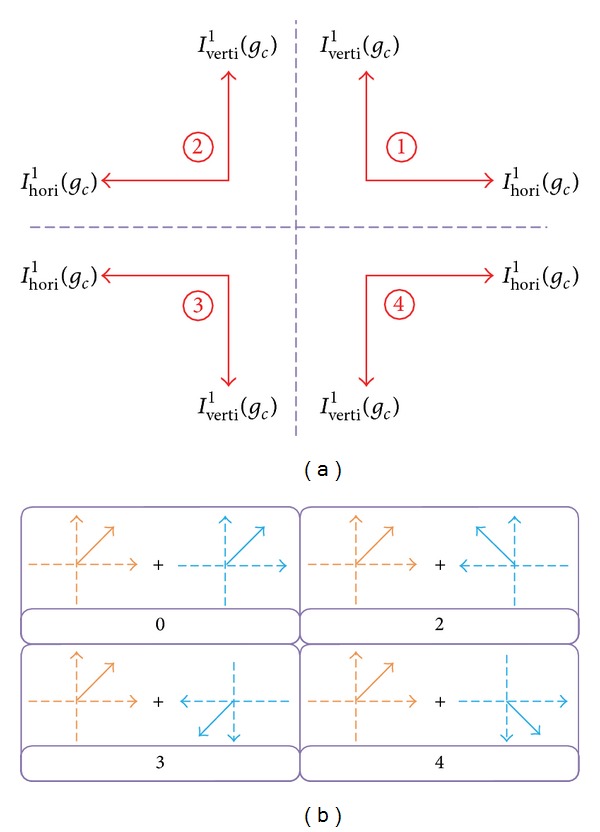
Illustrations tetra pattern based on one of four possible directions of each center pixel. (a) The directions of the center pixel based on (7). (b) Example of calculation of tetra pattern (see (8)-(9)) in the case of the center-pixel direction “1” defined from (7) using the direction of neighbors. Light wheat represents the direction of the center pixel and cyan represents its neighborhood pixels.

Tetra pattern is captured based on first-order derivatives and transformation consistency statistics of directional derivative. Given one image *I*, the first-order derivatives along horizontal and vertical direction at center pixel *g*_*c*_ can be written as

(6) $$\begin{array}{c} I_{\text{hori}}^{1}\left(g_{c}\right)=I\left(g_{k\_\text{hori}}\right)-I\left(g_{c}\right)\\ I_{\text{verti}}^{1}\left(g_{c}\right)=I\left(g_{k\_\text{verti}}\right)-I\left(g_{c}\right) \end{array}$$

and it is evident that the possible direction of each center pixel can be converted into (see Figure 2(a))

(7) $$\begin{array}{c} I_{\text{Dir}}^{1}\left(g_{c}\right)=\{\begin{array}{cc} 1, & I_{\text{hori}}^{1}\left(g_{c}\right)\geq 0,\;\;I_{\text{verti}}^{1}\left(g_{c}\right)\geq 0\\ 2, & I_{\text{hori}}^{1}\left(g_{c}\right)<0,\;\;I_{\text{verti}}^{1}\left(g_{c}\right)\geq 0\\ 3, & I_{\text{hori}}^{1}\left(g_{c}\right)<0,\;\;I_{\text{verti}}^{1}\left(g_{c}\right)<0\\ 4, & I_{\text{hori}}^{1}\left(g_{c}\right)\geq 0,\;\;I_{\text{verti}}^{1}\left(g_{c}\right)<0. \end{array} \end{array}$$

Then tetra pattern for each pixel can be captured as follows:

(8) LTrP2(gc)  ={f(IDir1(gc),IDir1(g0)),f(IDir1(gc),IDir1(g1)),…,f(IDir1(gc),IDir1(gN−1))},

(9) $$\begin{array}{c} f\left(I_{\text{Dir}}^{1}\left(g_{c}\right),I_{\text{Dir}}^{1}\left(g_{k}\right)\right)=\{\begin{array}{cc} 0, & I_{\text{Dir}}^{1}\left(g_{c}\right)=I_{\text{Dir}}^{1}\left(g_{k}\right)\\ I_{\text{Dir}}^{1}\left(g_{k}\right), & \text{otherwise}, \end{array} \end{array}$$

where *N* represents the number of neighboring pixels and the superscript is defined as “2” because (8) involves *N* 1-order functions.

**Figure 3:**
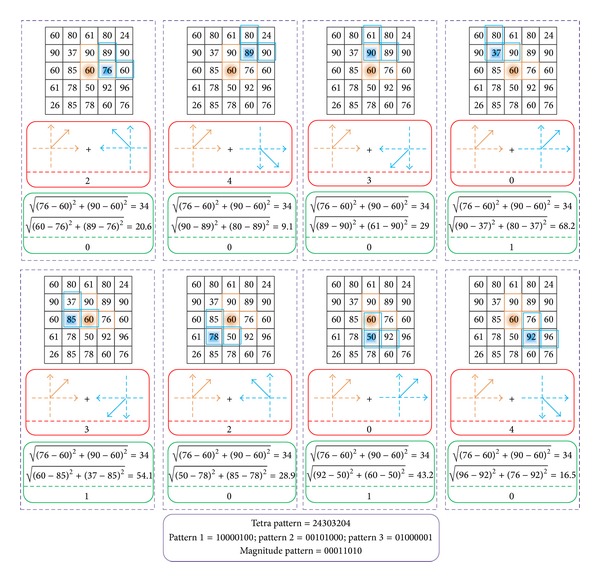
Example to obtain the tetra and magnitude patterns. For generating tetra pattern, the bit is coded with the direction of neighbor when the direction of the center pixel and its neighbor are different, otherwise “0,” which are represented in red rounded rectangle. Then convert the tetra patterns for each direction to three binary patterns, that is, “Pattern 1” to “Pattern 3” in bottom purple rounded rectangle. For generating the magnitude pattern, which is defined in (11), the bit is coded with “1” when the magnitude of the center pixel is less than its neighbor, otherwise “0,” and the binary magnitude coding is given in bottom purple rounded rectangle.

Take 8 neighborhoods into account and 8-bit tetra pattern for each center pixel. The possible values of the 8-bit of (8) are making up of four patterns, one is pattern 0 in (9), and the rest patterns are 3 of 4 patterns defined in (7). For example, taking *I*_Dir_^1^(*g*_*c*_) = 1 as an example, the possible values of the 8-bit of equation (8) are 0, 2, 3 and 4, see Figure 2(b). Also, taking *I*_Dir_^1^(*g*_*c*_) = 2 as another example, the possible values of the 8-bit of equation (8) are 1, 0, 3 and 4. The pattern “0” means *I*_Dir_^1^(*g*_*c*_) = *I*_Dir_^1^(*g*_*k*_), which is defined in equation (9). Finally, the tetra patterns are converted to three binary patterns. Take the direction of center pixel *I*_Dir_^1^(*g*_*c*_) to be equal to “1” in (7) as an example, then LTrP binary coding can be defined by setting 2, 3, and 4 to “1,”respectively, and the rest bits are set to “0”; see Figure 3. Finally, the binary coding of tetra patterns can be defined by segregating into three binary patterns as follows:

(10) $$\begin{array}{c} {\text{LTrP}}^{2}|{}_{\text{Dir}=2,3,4}=\overset{N-1}{\underset{k=0}{\sum }}2^{k}\cdot B\left({\text{LTrP}}^{2}\left(g_{c}\right)\right)|{}_{\text{Dir}=2,3,4}\\ B\left({\text{LTrP}}^{2}\left(g_{c}\right)\right)|{}_{\text{Dir}=d}=\{\begin{array}{cc} 1, & {\text{LTrP}}^{2}\left(g_{c}\right)=d\\ 0, & \text{otherwise}, \end{array} \end{array}$$

where *B*(·) represents binary conversion function and *d* stands for the direction of the center pixel; that is, *d* = 2, 3, 4 in the case of *I*_Dir_^1^(*g*_*c*_) = 1.

In a similar way, the other three tetra patterns for the remaining three directions in (8) can be converted to binary patterns. Hence, a total of 12 binary tetra patterns coding (4 ∗ 3, i.e., three binary tetra patterns in each four directions of center pixel) can be captured.

Intuitively, LBP can be understood as only sign pattern component of CLBP operators. Inspired by [15], to apply the combination of sign and magnitude components can provide better performance in classification. The 13th binary pattern coding (named LP in [14]) is captured by using the magnitude of horizontal and vertical first-order derivatives as follows (see Figure 3):

(11) $$\begin{array}{c} M_{I_{\text{Dir}}^{1}(g_{c})}=\sqrt{{\left(I_{\text{hori}}^{1}\left(g_{c}\right)\right)}^{2}+{\left(I_{\text{verti}}^{1}\left(g_{c}\right)\right)}^{2}}\\ \text{LP}=\overset{N-1}{\underset{k=0}{\sum }}2^{k}\cdot s\left(M_{I_{\text{Dir}}^{1}(g_{k})}-M_{I_{\text{Dir}}^{1}(g_{c})}\right), \end{array}$$

where *s*(·) represents sign function and is defined in (1).

In a nutshell, for generating binary tetra pattern, the bit is coded by the direction of neighbor when direction transformation is inconsistent between center pixel and its neighbors, and otherwise is coded with “0”; see (9); for generating binary magnitude pattern, the bit is coded with “0” when the magnitude of the center pixel is larger than that of its neighbor, and otherwise it is coded with “1.” Figure 3 gives an example to capture the binary tetra and magnitude patterns. In order to achieve a fair comparison to CLBP and LBP, only second-order tetra patterns LTrP^2^(*g*_*c*_) based on the same configurations of *R* = 1 and *N* = 8 are employed in this study and then converted the binary coding of tetra and magnitude pattern (for example, see the bottom of Figure 3) to decimal numeral. Finally computes the histogram of decimal coding to generate LTrP feature vectors based on some specified pattern mappings, and for more details about LTrP, for example, high-order tetra patterns, readers are suggested to read [14]. Hence, a total of 767- (59 ∗ 13) dimensional features, where 59 denotes the dimension after uniform pattern mapping and 13 denotes the number of binary patterns mentioned above, are employed to describe IHC images in this study.

#### 2.3.3. Feature Selections

In the field of machine learning and statistics, feature selection is defined as the process of selecting a subset of relevant features for use in subsequent model construction. Tremendous previous studies have demonstrated the necessity of feature selection because highly redundant and irrelevant feature sets should have an intrinsic dimensionality much smaller than the actual dimensionality of the original feature space [28–32]. Redundant features are those which provide no more information than the currently selected features, and irrelevant features provide no useful information in original feature space or time space context. Hence, in this study we applied stepwise discriminant analysis (SDA) approach, which used Wilk's *λ* statistic to iteratively determine the most discriminating feature subset in the feature space to feed into subsequent classifiers, and for more details about SDA, readers are suggested to read [12, 33]. Basically, three main benefits of feature selection techniques while constructing predictive models can be summarized as follows: (1) improved model interpretability, (2) shorter training times, and (3) enhanced generalization by reducing overfitting.

Two global features (i.e., Haralick texture and DNA distribution features) and three local pattern features (i.e., LBP, CLBP, and LTrP features) were employed to describe IHC images in this study. Three kinds of the combination of global and local features are fed into SDA for feature selection, which demonstrates the importance of all these features. According to our experimental results, more discriminative feature subset are selected, respectively, from various combination of global and local features and has played a positive role for analyzing the validity of the features. More details will be discussed in next section.

### 2.4. Classification Algorithm Level

As a multilabel classification algorithm, binary relevance (BR) method [18] is employed to train models in this study. Generally, there are two main methods for tackling the multilabel classification problem: problem transformation methods and algorithm adaptation methods. The former method transforms the multilabel problem into a set of binary classification problems, which can then be handled using single-class classifiers, for example, BR method. The latter method adapts the algorithms to directly perform multilabel classification, which is not the focus of this study. In BR models, the method called “cross-training” was used in training phase, which means each label is corresponding to its own classifier. For each classifier, all the training samples associated with the corresponding label are considered as positive samples while the rest as negative samples [34]. We applied support vector machine (SVM) along with LIBSVM toolbox (http://www.csie.ntu.edu.tw/~cjlin/libsvm/) to construct each of our BR classifier, where the parameters are obtained through brute force grid searching and 10-fold cross-validation in training phase [35].

### 2.5. Classification Evaluation Criteria and Multilabel Decision

In this paper, five multilabel classification evaluation metrics used in our previous works [11] are applied to evaluate the performance with twofold cross-validation. The five evaluation metrics are subset accuracy, accuracy, recall, precision, and average label accuracy, which are defined as follows.

Suppose that there are *L* classes and *q* testing samples, and let ${\hat{Y}}_{t_{j}}=[{\hat{y}}_{1}^{j},{\hat{y}}_{2}^{j},\ldots ,{\hat{y}}_{L}^{j}]$ denote the predicted label vector of the *j*th test sample *t*_*j*_, while *Y*_*t*_*j*__ = [*y*_1_^*j*^, *y*_2_^*j*^,…, *y*_*L*_^*j*^] is the corresponding true label vector.

(i)* Subset accuracy* is the fraction of samples, whose predicted label set is identical with the true label set.

Consider

(12) $$\begin{array}{c} \text{Subset}\;\text{accuracy}=\frac{1}{q}\overset{q}{\underset{j=1}{\sum }}\Phi ⟦{\hat{Y}}_{t_{j}}=Y_{t_{j}}⟧, \end{array}$$

where

(13) $$\begin{array}{c} \Phi ⟦\cdot ⟧=\{\begin{array}{cc} 1, & \cdot \;\;\text{is}\;\;\text{true}\\ 0, & \text{otherwise}. \end{array} \end{array}$$

(ii)* Accuracy* is more lenient to errors than subset accuracy because if not all the predicted labels of a sample are correct, then subset accuracy gives 0, but accuracy gives a value between 0 and 1, reflecting the degree of partial correctness.

Consider

(14) $$\begin{array}{c} \text{Accuracy}=\frac{1}{q}\overset{q}{\underset{j=1}{\sum }}\text{point}\left({\hat{Y}}_{t_{j}}\right). \end{array}$$

Each test sample prediction can be scored by

(15) $$\begin{array}{c} \text{point}\left({\hat{Y}}_{t_{j}}\right)=\frac{\sum_{l=1}^{L}\Phi ⟦y_{l}^{j}=1,{\hat{y}}_{l}^{j}=1⟧}{\sum_{l=1}^{L}\Phi ⟦y_{l}^{j}=1,\text{or}\;\;{\hat{y}}_{l}^{j}=1⟧}. \end{array}$$

(iii)* Recall* is the fraction of true labels that are correctly predicted in testing phase and can be considered as an extension of the classic definition to measure recall of each class in conventional single-label learning.

For a class *l*, *l* = 1,2,…, *L*,

(16) $$\begin{array}{c} \text{Recall}\left(l\right)=\frac{1}{\sum_{j=1}^{q}\Phi ⟦y_{l}^{j}=1⟧}\underset{t_{j}\in \left\{t_{j}\;|\;y_{l}^{j}=1\right\}}{\sum }\text{point}\left({\hat{Y}}_{t_{j}}\right). \end{array}$$

Then the uniform recall of the total testing samples is computed as

(17) $$\begin{array}{c} \text{Recall}=\frac{1}{L}\overset{L}{\underset{l=1}{\sum }}\text{Recall}\left(l\right). \end{array}$$

(iv)* Precision* is the fraction of predicted labels that are correctly predicted and also can be considered as an extension of the classic definition to measure precision of each class in conventional single-label learning.

For a class *l*, *l* = 1,2,…, *L*,

(18) $$\begin{array}{c} \text{Precision}\left(l\right)=\frac{1}{\sum_{j=1}^{q}\Phi ⟦{\hat{y}}_{l}^{j}=1⟧}\underset{t_{j}\in \left\{t_{j}\;|\;{\hat{y}}_{l}^{j}=1\right\}}{\sum }\text{point}\left({\hat{Y}}_{t_{j}}\right). \end{array}$$

Then the uniform precision of the total testing samples is computed as

(19) $$\begin{array}{c} \text{Precision}=\frac{1}{L}\overset{L}{\underset{l=1}{\sum }}\text{Precision}\left(l\right). \end{array}$$

(v)* Label accuracy* evaluates the prediction accuracy for each label and devotes to identify which subcellular locations are easier to recognize.

For a class *l*, *l* = 1,2,…, *L*,

(20) $$\begin{array}{c} \text{Label}\;\text{accuracy}\left(l\right)=\frac{1}{q}\overset{q}{\underset{j=1}{\sum }}⟦{\hat{y}}_{l}^{j}=y_{l}^{j}⟧. \end{array}$$

Then the average label accuracy computes the average of *L* accuracies of labels and can reflect the total performance.

Consider

(21) $$\begin{array}{c} \text{Average}\;\text{label}\;\text{accuracy}=\frac{1}{L}\overset{L}{\underset{l=1}{\sum }}\text{Label}\_\text{accuracy}\left(l\right). \end{array}$$

Since six major organelles were concerned in this study, each training model is corresponding to six classifiers and each classifier will output a score from an independent SVM. Namely, each model based on BR outputs a 6-dimensional score vector, where each score represents the confidence of belonging to a specific label. Then, a threshold strategy is employed to decide the label set of a sample from its score vector outputs. In detail, for *j*th test sample whose score vector output is defined as [*O*_1_^*j*^, *O*_2_^*j*^,…, *O*_*L*_^*j*^], and then the elements of final label decision ${\hat{Y}}_{t_{j}}=[{\hat{y}}_{1}^{j},{\hat{y}}_{2}^{j},\ldots ,{\hat{y}}_{L}^{j}]$ (denotes the predicted label vector of the *j*th test sample *t*_*j*_) are defined as follows:

(22) $$\begin{array}{c} {\hat{y}}_{i}^{j}=\{\begin{array}{cc} 1 & O_{i}^{j}\geq T\\ -1 & \text{otherwise}, \end{array} \end{array}$$

where *i* = 1,2,…, *L* and *j* = 1,2,…, *q*, and *T* denotes the threshold.

The threshold strategy considers the final label set that is composed by the labels whose scores are larger than the threshold *T*. In this study, we applied grid search approach from −2 to 2 to find an optimal threshold by cross-validation tests, which is determined by maximizing the subset accuracy evaluation index [11]. The subset accuracy, which denotes the fraction of samples whose predicted label set is exactly matched with the true label set, is the most important evaluation index among all performance of classification in this study. However, one weakness of threshold strategy would be revealed and obviously will reduce the authenticity of subset accuracy index. Namely, a special situation of no label assignment might occur when simply using threshold strategy because it is possible that all scores are less than the threshold. To overcome this obstacle, a guarantee strategy is employed based on threshold strategy; that is, the highest score wins when all of the scores are less than the threshold and corresponding single label is assigned to the sample. Hence, the combination of threshold strategy and guarantee strategy are employed in this study to confirm the impartiality and effectiveness of subset accuracy evaluation index.

## 3. Experimental Results and Discussions

### 3.1. Results on Influence by Local Pattern Features for IHC Images

Generally, efforts for developing effective image descriptors for AI-PSLP can be summarized into the following two categories: conventional global distribution features and local pattern features. Since the IHC images are characterized by high quality and resolution, more effective local descriptors are required to capture more effective local pattern information. Hence, besides the widely used global features, that is, Haralick texture feature and DNA-protein overlap feature, both CLBP and LTrP are investigated to describe IHC images for the first time and applied to AI-PSLP. The dimension of the global feature set is 840 (836 + 4), whose details are described in Section 2.3.1, and the local pattern feature set involves 256-dimensional LBP features, 200-dimensional CLBP features, and 767-dimensional LTrP features, whose details are described in Section 2.3.2. We systematically analyzed the outputs from SDA feature selection, and three conclusions can be summarized as follows.

Firstly, the proportion of local pattern features increases while that of global features declines after SDA feature selection, which means the local pattern features play a positive complementary role to the global features.  Table 1 shows the proportion of different local pattern features incorporating with global features. For example, the proportion of SLFs_CLBP (symbol “_” denotes combination) features is ~19% (200/1040) in original feature set and rises to approximately 42% (the average proportion of all folds in Table 1) after SDA feature selection, which means the redundancy of local pattern feature is less than the global features, and the combination of CLBP and global feature are positive and significant. Similar rule can be found in LTrP and LBP: the proportion of SLFs_LTrP features rises from 48% (767/1607) to 66% (also the average proportion of all folds in Table 1), and the proportion of SLFs_LBP features rises from 23% (256/1096) to 50% through SDA. The difference of the proportion of three local pattern features in original feature sets is due to different pattern mapping strategies.

Secondly, the rank of local pattern features is higher, which demonstrates again the vital importance of the local pattern features.  Table 2 shows the feature ranks obtained by SDA for all these three local pattern features incorporating with db6 global features in each fold. Generally, the higher feature rank obtained from SDA, the more discriminative characteristic of feature stands for. As shown in Table 2, for each combination of local and global features, the local pattern feature received a good ranking order in top 15 rankings. More strictly in Table 2, taking the top 5 for comparison, CLBP has won at most 3 seats of top 5, and LTrP has won at most 2 seats, and LBP has won only 1 seat, which means CLBP is the most effective descriptor for IHC images among all the three local pattern descriptor. This is because the CLBP descriptor has concerned three aspects, that is, sign level, magnitude level, and center pixel level. Although the consistency of the gradual change in horizontal and vertical directions between the center pixel and its neighbors is concerned by LTrP descriptor, essentially, LTrP belongs to a variety descriptor of sign level incorporating with magnitude, that is, a more complicated sign-magnitude-based descriptor. Namely, LTrP only considers the direction change and magnitude statistics and ignores the most fundamental information, the value of original center pixel. Obviously, the standard LBP only considers the local region sign-liked statistics, which means ignoring both the local magnitude pattern and center pixel gray value pattern statistics.

Thirdly, for each combination of local pattern features and global features, the performance of AI-PSLP has been improved shown in Table 3. Although the more discriminative feature subset is obtained due to excellent local pattern descriptors being fed into SDA, the ultimate effectiveness of these local descriptors must be validated by the subsequent classification results, where details are given as follows.

### 3.2. Results on Binary Relevance Models and Enhanced by Threshold Strategy and Guarantee Strategy

The method called “cross-training” [34] was employed for multilabel benchmark to construct models in training phase based on support vector machine (SVM). Six major subcellular locations are concerned in this study, each training model is corresponding to six classifiers, and the outputs of each of six independent SVM classifiers represent the confidence of a sample belonging to a specific label. All results were obtained using twofold cross-validation in our benchmark, and we performed twofold cross-validation in a protein-based approach. Namely, images of any protein that appear in the training set will not appear in the testing set. In other words, the testing set can be considered as an independent set relative to training set. In testing phase, threshold strategy together with guarantee strategy is employed to finally decide the label set of a sample (both single-label and multilabel) from the corresponding 6-dimensional score vector. Table 3 shows the comparison of the two scenarios based on threshold strategy and guarantee strategy, and two conclusions can be summarized as follows.

Firstly, the performances of SLFs_LTrP (symbol “_” denotes combination) and SLFs_CLPB are better than SLFs_LBP. Since subset accuracy denotes the fraction of samples whose predicted label set is exactly matched with the true label set, which is the most important evaluation index among all performance of classification in this study. From Table 3, the comparisons of subset accuracy among the three local pattern features combining with global features are given. Although the threshold strategy can deal with our multilabel benchmark, the subset accuracies are very low, for example, the average subset accuracies of SFLs_LBP, SFLs_CLBP, and SFLs_LTrP based on threshold strategy are 33%, 37%, and 35%, respectively. (data not shown) Obviously, the performance by using both of the two novel local pattern descriptors, that is, LTrP and CLBP, is a little better than standard local binary but still seems very low. This is because lots of samples are not assigned label due to the weakness of threshold strategy, and this also motivates us to carry out our works in circumstance of more reasonable statistics, that is, based on guarantee strategy.

Secondly, all results of the combination between local pattern feature and global feature have been improved by using the guarantee strategy based on original threshold strategy. In Table 3, the subset accuracy of SLFs_LBP (denotes the combination of global feature and standard local binary pattern) is improved from the original 33% to 42%, and the subset accuracy of SLFs_CLBP is improved from 37% to 46%, and the subset accuracy of SLFs_LTrP is improved from 35% to 44%. All these percentages denote the average of all folds from db1 to db10. Each value in Table 3 refers to the average subset accuracy of two folds in each db, and boldface type in Table 3 denotes the maximum value of each row.

In general, not only more discriminative feature subset is obtained due to excellent local pattern descriptors are being fed into SDA, but also the ultimate effectiveness of these local descriptors have been demonstrated by the subsequent classification results.

### 3.3. Results on Multilabel Samples

**Figure 4:**
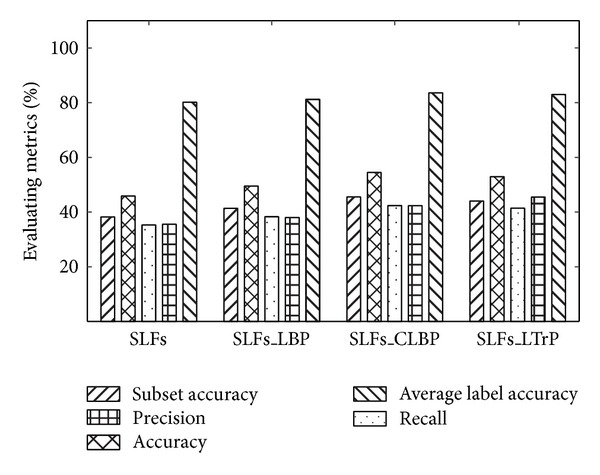
The comprehensive evaluation of entire dataset (348 proteins) based on db6 global features and local pattern features.

A comprehensive evaluation of entire dataset (348 proteins) is shown in Figure 4, and all the evaluation indexes correspond to the average of twofold in combination of db6 global features and local pattern features. In a nutshell, all results in Figure 4 are based on the same configuration except different combination of global and local pattern features. In order to demonstrate the importance of local pattern features, the evaluation indexes based solely on global feature are also given for comparison. Two conclusions can be summarized as follows.

Firstly, the local pattern features are demonstrated by not only SDA in feature level, but also the ultimate effectiveness of LTrP and CLBP descriptors that have been demonstrated by the classification results (five evaluation indexes). From Figure 4, the results show that all of the five evaluation indexes are improved. For instance, the subset accuracy of SLFs_CLBP is improved saliently among three different combinations of global and local pattern features while compared to those situations of SFLs and accuracy evaluation index also improved significantly. However, only the importance of local pattern features can be demonstrated form Figure 4, the improvement range comparison whether dataset are involved with multilabel samples is not shown.

Secondly, the evaluation indexes of BR model feeding by more discriminative local features, such as LTrP and CLBP, can always remain high growth in both single-label and multilabel datasets. Five evaluation index comparisons are shown in Table 4 based on single-label (258 proteins) and entire dataset (348 proteins) by using BR model fed into different combinations of local and db6 global features. In this study, we also applied protein-based twofold cross-validation approach to perform on single-label dataset and to ensure that there were never any images in training and testing phase from the same protein. When focus on single-label dataset, the improvement of the subset accuracy by employing CLBP together with global features rises by 8.14% (from 50.58% to 58.72%) when compared to using global features alone. The improvement of the subset accuracy by employing LTrP features together with global features is also higher than the SLFs by 6.87%, a little bit less than that of CLBP. The improvement of SLFs_LBP is the least, that is, 5.17%.

When evaluated on the single-label samples together with multilabel samples, the evaluation indexes can be maintained at a reasonable level of growth. For instance, the improvements of the subset accuracy are 7.3%, 5.8%, and 3.15%, while employing global features together with CLBP, LTrP, and LBP, respectively (all compared to the traditional global SLFs features). The different ratios of improvement indicate that CLBP and LTrP are better for image-based multilabel human protein subcellular localization classification than LBP. Similarly, the other four evaluation indexes can give the same conclusion from Table 4.

### 3.4. Discussions

As a subfield of bioinformatics and computational biology, bioimage informatics focuses on the use of image processing and computational techniques to analyze bioimages, especially subcellular images. Features of AI-PSLP in early stage are either generic features (e.g., Haralick texture feature) or features specially designed to capture biological factors (e.g., colocalization with a nuclear marker being a typical example, called DNA overlap feature). Both these features belong to global features. However, a few local pattern features have attracted much attention in the field of bioimage informatics because of the robustness against rotation and translation in localized regions of images.

To our knowledge, two types of these local pattern features can be summarized and used in the field of bioimage informatics. The first type of local pattern features apply local structural model quantization between center pixel and its neighborhoods, such as standard LBP [25, 26] and its variants (e.g., LTP [10], LQP [9], LTCoP [36], and CLBP [15]). In other words, the essence of this kind of local pattern descriptor focuses on the gradient changes of the center pixel in specified directions. The second type of local pattern features focuses on transformation consistency statistics of directional derivative in specified directions between the center pixel and its neighbors (e.g., LDP [37] and LTrP [14]).

Table 5 summarizes the local pattern features that can be applied to analyze the bioimages. Two local pattern features, each of which coming from one of two types of local pattern features mentioned above, are investigated and compared to standard LBP feature in this study. According to our experimental results, we found both of the two local pattern operators to have their own advantages. Compared to the standard LBP descriptor, both the CLBP and the LTrP are able to generate better results. The reasons are as follows.

1. For the CLBP descriptor, it has gotten a better performance because of taking additional information into account, that is, magnitude level and center pixel level.
2. For the LTrP descriptor, it is able to generate a better performance due to more detailed statistics in IHC image, that is, not only including the statistics on gradient changes of the center pixel in specified directions, but also statistics on the transformation consistency of directional derivative in specified directions between the center pixel and its neighbors at the cost of paying more computational time.

Hence, how to describe a bioimage covering both of the above two advantages will be an interesting future effort.

## 4. Conclusions and Future Work

We have investigated LTrP and CLBP to describe IHC images for the first time and applied it to AI-PSLP, and much advantage of employing both of the two local pattern descriptors have been demonstrated in our experimental results to be embodied not only in feature selection level, for example, the redundancy of local pattern features is less than those of global features, but also in the enhanced performance of subsequent classification.

For more precise interpretation of effective information to key positions of images, both the changes of gradient direction and magnitude of the corresponding neighborhood pixels of the center pixel are necessarily considered. However, how to capture and encode these changes between the center pixel and its neighbors are crucial. LBP can be seen as an approach of traditionally divergent statistics between center pixel and its neighbors. CLBP descriptor can be considered as the fusion of capturing three aspects of changes between the center pixel and its neighbors, and the three aspects of changes are named sign level, magnitude level, and center pixel level. On the other hand, LTrP can be considered as a variety descriptor of sign level incorporating with magnitude, that is, a more complicated sign-magnitude-based descriptor.

Since all the CLBP, LTrP, and LBP are used to describe the local texture features of an image, the reason why our experimental results show the CLBP is better than the others can be that CLBP is capable of capturing the symbol information together with magnitude and center pixel level information at the same time, which is the most distinguishing trait compared to LBP; and it is also able to capture center pixel level information, which is different from the LTrP. In summary, the CLBP can describe more IHC image local patterns and thus can be able to derive better results.

Many future works are expected to be carried out, for example, the influence of local pattern mapping (uniform and rotation invariance pattern mapping), the trade-off between the effectiveness of high-order directional derivative (high-order LTrP) and the complexity. Moreover, in order to be qualified for multilabel benchmark classification, better multilabel learning and label decision models are required to further improve the multilabel samples classification accuracy.

## Figures and Tables

**Table 1:** Comparison SDA results derived from the combination of different local pattern features and global features.

DB	Fold	1096 dimensional SLFs_LBP feature fed into SDA		1040 dimensional SLFs_CLBP feature fed into SDA		1607 dimensional SLFs_LTrP feature fed into SDA	
db1	fold 1	55 (D1-H30-L24)	44%	50 (D1-H25-C24)	48%	69 (D2-H21-T46)	67%
	fold 2	48 (D3-H22-L23)	48%	40 (D2-H19-C19)	48%	59 (D3-H22-T34)	58%

db2	fold 1	66 (D1-H39-L26)	39%	49 (D1-H32-C16)	33%	70 (D2-H29-T39)	56%
	fold 2	49 (D3-H21-L25)	51%	42 (D3-H23-C16)	38%	52 (D2-H17-T33)	63%

db3	fold 1	58 (D2-H28-L28)	48%	50 (D1-H27-C22)	44%	66 (D2-H20-T44)	67%
	fold 2	34 (D3-H13-L18)	53%	44 (D3-H23-C18)	41%	45 (D2-H12-T31)	69%

db4	fold 1	53 (D1-H28-L24)	45%	53 (D2-H27-C24)	45%	69 (D22-H3-T44)	64%
	fold 2	39 (D1-H16-L22)	56%	42 (D2-H24-C16)	38%	48 (D1-H16-T31)	65%

db5	fold 1	53 (D1-H25-L27)	**51%**	49 (D2-H27-C20)	41%	64 (D1-H24-T39)	61%
	fold 2	44 (D3-H15-L26)	59%	42 (D3-H19-C20)	48%	46 (D1-H11-T34)	74%

db6	fold 1	49 (D1-H22-L26)	53%	46 (D2-H23-C21)	46%	68 (D3-H20-T45)	**66%**
	fold 2	40 (D3-H22-L15)	38%	46 (D3-H27-C16)	35%	45 (D1-H13-T31)	66%

db7	fold 1	56 (D1-H32-L23)	41%	49 (D1-H28-C10)	20%	65 (D3-H19-T43)	66%
	fold 2	39 (D3-H18-L18)	46%	41 (D2-H17-C22)	54%	42 (D1-H12-T29)	69%

db8	fold 1	53 (D1-H26-L26)	49%	44 (D2-H23-C19)	43%	61 (D1-H17-T43)	70%
	fold 2	40 (D2-H14-L24)	60%	42 (D2-H21-C19)	45%	51 (D1-H13-T37)	73%

db9	fold 1	54 (D1-H29-L24)	44%	51 (D3-H31-C17)	33%	67 (D3-H23-T41)	61%
	fold 2	43 (D2-H17-L24)	56%	43 (D3-H23-C17)	40%	54 (D2-H16-T36)	67%

db10	fold 1	55 (D1-H24-L30)	55%	45 (D1-H25-C19)	**42%**	65 (D2-H23-T40)	62%
	fold 2	46 (D2-H17-L27)	59%	47 (D3-H19-C24)	51%	52 (D2-H15-T35)	67%

D, H, L, C, and T denote DNA-protein overlap feature, Haralick feature, LBP, CLBP, and LTrP, respectively. DB denotes 10 different lengths of vanishing moment of Daubechies wavelet. All percentages denote the proportion of local pattern features in the whole feature set, and boldface type denotes the value closest to the mean of that column.

**Table 2:** An illustration of features rank obtained by SDA for the combination of each of three local pattern features and global features in db6 of training set of each 2-fold.

Rank		1	2	3	4	5	6	7	8	9	10	11	12	13	14	15
SLFs	f1	H	D	H	H	H	H	H	H	H	H	H	H	H	H	H
	f2	D	H	H	H	H	H	H	H	H	H	H	H	H	H	D

SLFs_LBP	f1	H	D	H	H	L	L	L	L	H	L	H	L	L	L	L
	f2	D	H	H	H	L	L	L	H	H	H	H	H	L	L	H

SLFs_LTrP	f1	H	T	D	T	H	H	T	T	T	T	T	T	H	H	T
	f2	D	H	T	H	H	T	T	T	T	T	T	T	T	H	H

SLFs_CLBP	f1	C	H	C	D	C	H	H	C	C	C	C	C	H	H	H
	f2	D	C	H	H	H	H	H	C	C	H	C	H	D	C	H

f1 and f2 denote each fold of 2-fold cross-validation. H denotes Haralick feature, D denotes DNA-protein overlap feature, L denotes LBP feature, T denotes LTrP feature, and C denotes CLPB feature.

**Table 3:** Comparison of subset accuracies with different local pattern features.

Feature combination	Subset accuracy									
	db1	db2	db3	db4	db5	db6	db7	db8	db9	db10
SLFs	0.3715	**0.3899**	0.3862	0.3864	0.3719	0.3825	0.3678	0.3749	0.3832	0.3699

SLFs_LBP	0.4170	**0.4269**	0.4134	0.4194	0.4111	0.4140	0.4065	0.4088	0.4136	0.4210

SLFs_CLPB	0.4463	0.4362	0.4394	0.4481	0.4334	**0.4556**	0.4378	0.4398	0.4513	0.4449

SLFs_LTrP	0.4368	0.4399	0.4283	0.4385	**0.4438**	0.4405	0.4285	0.4323	0.4354	0.4339

Boldface type denotes the maximum value of each row, which corresponds to the best average subset accuracy of two fold in each db.

**Table 4:** Five evaluation index comparisons based on single-label and entire dataset by using BR model fed into different combinations of local and db6 global features.

Evaluation index	Single label samples (258 proteins)				Entire dataset (348 proteins)			
	SLFs	SLFs_LBP	SLFs_CLBP	SLFs_LTrP	SLFs	SLFs_LBP	SLFs_CLBP	SLFs_LTrP
Subset accuracy	0.5058	0.5575	0.5872	0.5745	0.3825	0.4140	0.4555	0.4405
Accuracy	0.5141	0.5713	0.6153	0.5975	0.4586	0.4956	0.5451	0.5296
Recall	0.3855	0.4159	0.4640	0.4198	0.3528	0.3831	0.4240	0.4141
precision	0.3697	0.3944	0.4706	0.4610	0.3555	0.3802	0.4233	0.4552
Label accuracy	0.8353	0.8492	0.8508	0.8452	0.8020	0.8124	0.8360	0.8302

258 proteins correspond to single-label samples in our benchmark dataset, and 348 proteins denote entire dataset involved with single-label and multilabel samples.

All columns correspond to average of 2-fold on db6. SLFs denote global features involved with Haralick features and DNA-protein overlap features. Label accuracy denotes the average prediction accuracy of six labels.

**Table 5:** Summarization of local pattern features focusing on bioimage informatics studies.

Number	Name of local pattern features	Brief description	Types
1	*Local binary pattern (LBP) *	A pioneer of local structural model quantization and the concatenate histogram statistics.	Type 1: focus on the gradient changes of the center pixel in specified directions.
2	Local ternary pattern (LTP)	Using a ternary arithmetic coding given a threshold based on LBP.	
3	Local quinary pattern (LQP)	Two thresholds enhancement based on LTP.	
4	*Completed local binary pattern (CLBP) *	Enhance by taking magnitude and center pixel level information into account based on Type 1.	
5	Local ternary cooccurrence pattern (LTCoP)	Encodes the cooccurrence of similar ternary edges calculated between the center pixel and its neighbors based on LTP; belongs to rotational invariant feature.	

6	Local derivative pattern (LDP)	High-order local pattern descriptor and encodes directional pattern features based on local derivative variations; belongs to a specific direction rotational variant feature.	Type 2: focus on transformation consistency statistics of directional derivative in specified directions between the center pixel and its neighbors.
7	*Local tetra pattern (LTrP) *	Encodes the relationship of transformation consistency between the center pixel and its neighbors based on vertical and horizontal directions.	

Italic type denotes the local pattern features investigated in this study.
